# Alteration of the Microbiota and Virulence Gene Expression in *E*. *coli* O157:H7 in Pig Ligated Intestine with and without AE Lesions

**DOI:** 10.1371/journal.pone.0130272

**Published:** 2015-06-19

**Authors:** Bianfang Liu, Xianhua Yin, Hai Yu, Yanni Feng, Xin Ying, Joshua Gong, Carlton L. Gyles

**Affiliations:** 1 College of Food Science and Engineering, Northwest A&F University, Yangling, Shaanxi Province, P. R. China; 2 Guelph Food Research Centre, Agriculture and Agri-Food Canada, Guelph, Ontario, Canada; 3 College of Animal Science and Technology, Qingdao Agricultural University, Qingdao, P. R. China; 4 China National Cereals, Oils and Foodstuff s Corporation, Beijing, P. R. China; 5 Department of Pathobiology, Ontario Veterinary College, University of Guelph, Guelph, Ontario, Canada; University of Arizona, UNITED STATES

## Abstract

**Background:**

Previously we found that *E*. *coli* O157:H7 inoculated into ligated pig intestine formed attaching and effacing (AE) lesions in some pigs but not in others. The present study evaluated changes in the microbial community and in virulence gene expression in *E*. *coli* O157:H7 in ligated pig intestine in which the bacteria formed AE lesions or failed to form AE lesions.

**Methodology/Principal Findings:**

The intestinal microbiota was assessed by RNA-based denaturing gradient gel electrophoresis (DGGE) analysis. The DGGE banding patterns showed distinct differences involving two bands which had increased intensity specifically in AE-negative pigs (AE- bands) and several bands which were more abundant in AE-positive pigs. Sequence analysis revealed that the two AE- bands belonged to *Veillonella caviae*, a species with probiotic properties, and *Bacteroides* sp. Concurrent with the differences in microbiota, gene expression analysis by quantitative PCR showed that, compared with AE negative pigs, *E*. *coli *O157:H7 in AE positive pigs had upregulated genes for putative adhesins, non-LEE encoded *nleA* and quorum sensing *qseF*, acid resistance gene *ureD*, and genes from the locus of enterocyte effacement (LEE).

**Conclusions/Significance:**

The present study demonstrated that AE-positive pigs had reduced activities or populations of *Veillonella caviae* and *Bacterioides* sp. compared with AE-negative pigs. Further studies are required to understand how the microbiota was changed and the role of these organisms in the control of *E*. *coli* O157:H7.

## Introduction

Enterohemorrhagic *Escherichia coli* (EHEC) O157:H7 is an enteric pathogen that causes foodborne disease ranging from uncomplicated diarrhea to hemorrhagic colitis (HC) and life-threatening hemolytic uremic syndrome (HUS) in humans [1]. Two major virulence factors are involved in causing disease: products of a pathogenicity island named the locus of enterocyte effacement (LEE), that are needed for intestinal colonization, and Shiga toxin (Stx) which causes damage to tissues [2]. The LEE encodes a type III secretion system which secretes proteins involved in signal transduction and subversion of host cell functions, and the adhesin molecule intimin (encoded by *eae*) and its receptor (Tir) required for intimate host-cell interaction [3]. LEE genes are required for the formation of the attaching and effacing (AE) lesion, which is characterized by localized destruction of microvilli and intimate attachment of bacteria to the apical enterocyte membrane [2, 4]. Additional putative virulence factors, such as putative adhesins Iha (IrgA homolog adhesin) and EhaA (EHEC autotransporter) [5, 6], and pO157 plasmid-encoded StcE [7], may also play roles in pathogenesis [8].

AE lesion formation and expression and secretion of LEE gene products are regulated by a variety of environmental clues, including nutrient availability, quorum sensing, global regulators such as IHF, FIS, H-NS and RpoS, and pathogen-specific regulators such as Ler [2, 5, 9]. However, little is known about regulation *in vivo*. We have shown that *E*. *coli* O157:H7 grown in MacConkey broth and exposed to pH 2.5 had little or no adherence to intestinal epithelial cells *in vitro*, but caused significant adherence and AE lesions when inoculated into pig intestinal loops [10, 11], suggesting that the intestinal environment, including the microbiota, favors the expression and secretion of virulence factors for infection and AE lesion [5, 10, 12]. This hypothesis is supported by the observations that EHEC O157:H7 caused more robust AE lesions on intestinal epithelial cells than on cultured cells [8, 13]. A healthy intestinal microbiota, a microbial community consisting of eukaryotes, viruses and bacteria, is essential for the health of the host and provides protection against enteric infection [14]. Alteration of the microbial community has been shown to influence susceptibility of the host to *Salmonella* infection [14, 15]. We have shown previously that there was a large variation in adherence in intestinal loops in littermate pigs challenged with EHEC O157:H7 bacteria; although the bacterial inocula were identical, some pigs developed AE lesions while others did not [16, 17]. It is critical to understand why certain pigs are more prone to colonization and AE lesion development by EHEC than others. Alteration of microbiota was hypothesized to be involved. Therefore, the present study analyzed effect of inoculated *E*. *coli* O157:H7 strain 86–24 on the metabolically active microbial population in the pig ligated ileum with or without AE lesion was evaluated by PCR-DGGE analysis of 16S rRNA genes, and compared the expression of major virulence factors and putative virulence genes of *E*. *coli* O157:H7 recovered from the pig ligated intestine with and without AE lesions.

## Materials and Methods

### Ethics statement

The experimental protocols and care of the animals were approved by the University of Guelph Animal Care Committee (Approval ID #05R143).

### Pig gut-loop experiments

*E*. *coli* O157:H7 strain 86–24 was grown in brain-heart infusion (BHI) broth plus NaHCO_3_ (final concentration 44 mM) (BHIN) at 37°C overnight statically, concentrated by centrifugation and resuspended in Eagle’s minimum essential medium (EMEM) containing 10% fetal bovine serum (FBS) to prepare an inoculum of approximately 5x10^10^ cfu/mL of bacteria.

A total of 34 female pigs (12 to 14 days old) were used, with two or three pigs from the same litter being used at one time; three of the pigs were used as control pigs. The pigs were obtained from the University of Guelph Swine Research Station. The pigs were housed together and had access to a balanced electrolyte solution (Vetoquinol, Lavaltrie, Quebec) but no food for 24 h before surgery. The pigs were premedicated with a mixture of ketamine (50 mg/mL), xylazine (10 mg/mL), and butorphenol (1 mg/mL), given intramuscularly at 0.2 mL/kg body weight. About 10 min later anesthesia was achieved by slow intravenous injection of sodium pentobarbital (55 mg/100 mL). Following cleaning and disinfection of the abdomen, a ventral midline laparotomy was performed aseptically and the distal ileum was exteriorized. Loops (each about 10 cm long) were created with nylon ligatures in the distal ileum, beginning approximately 10 cm from the ileocecal junction. Each loop was followed by a short intervening segment (2–3 cm) that was not inoculated. In each pig, 4 loops were used for this study; 2 loops were for *E*. *coli* O157:H7 grown in BHIN, and 2 loops for EMEM. The loops were randomly assigned and were inoculated with a 25 gauge needle; each received a 2-mL volume of either *E*. *coli* O157:H7 strain 86–24 (10^11^ CFU) or EMEM. After inoculation, the ileum was replaced in the abdomen and the laparotomy incision was closed. Immediately following the surgery and at 4-h intervals thereafter, the pigs were injected intramuscularly with butorphenol (Wyeth Canada, St. Lautent, QC) at 0.4 mg/kg body weight. The pigs were euthanized by an overdose of pentobarbital 15–16 h after inoculation of the loops. The ligated segments of intestine were quickly excised and the loop contents were collected in RNAlater at 1:5 ratio (Ambion, TX), kept at 4°C overnight and stored at -80°C until RNA isolation.

### Histological examination

Tissues taken from the loops were fixed immediately in 10% neutral buffered formalin at room temperature for at least 24 h. The fixed tissues were cut into smaller pieces (approximately 0.5x1.5 cm) and every second piece of tissue was chosen for a total of four pieces from each loop that were processed by routine methods. After embedding the tissue in paraffin 1 μm-thick sections were cut and stained with Giemsa. All villi in these sections were examined by light microscopy to determine the percentage of villi with adherent bacterial clusters (≥5 bacteria). Selected fixed tissues likely to contain AE lesions as identified by the light microscopic examination were processed for electron microscopy (EM). Thin sections were stained with uranyl acetate and lead citrate and examined with a 100S transmission electronmicroscope (Joel, Japan).

### RNA isolation

Ileal loop contents of 8 pigs with positive AE lesions and 4 pigs without AE lesions were selected for RNA isolation. In parallel, RNA was also isolated from the contents of the ileal loops from the 3 control pigs inoculated with EMEM.

Bacterial total RNA was isolated using the RiboPure-Bacteria kit protocol (Ambion, TX), with some modifications. Briefly, samples in RNAlater from pig loop contents were mixed with equal volumes of PBS and centrifuged at 5000 × *g* for 10 min. The bacterial pellets were resuspended in RNAwiz (lysis buffer), transferred to 2 mL screw-capped tubes containing ~500 μL Zirconia beads, and subjected to bead-beating by a Mini-Beadbeater (Bioscience Products) for 90 s twice with cooling on ice for 2 min in between. Subsequent steps for RNA isolation and purification and DNase I treatment were followed according to the manufacturer’s protocol. The extracted RNA was treated several times with DNase I until it was confirmed to be free of genomic DNA contamination, as determined by PCR using RNA as the template. Total RNA was quantified by a NanoDrop ND-1000 spectrophotometer (NanoDrop Technologies), and RNA quality was confirmed by visualization on agarose gel.

### Reverse transcription (RT)

First-strand cDNA was synthesized from a 1.0 μg quantity of the DNase I-treated bacterial total RNA using SuperScript II reverse transcriptase with 100 ng random primer pd(N)9 according to the procedures recommended by the supplier (Invitrogen, Carlsbad, CA, USA).

### Bacterial 16S rRNA transcript amplification and denaturing gradient gel electrophoresis (DGGE)

The V3 region of the 16S rRNA genes of Eubacteria was amplified using primers HDA1-GC (**CGC CCG GGG CGC GCC CCG GGC GGG GCG GGG GCA CGG GGG G**AC TCC TAC GGG AGG CAG CAG T-3’; the GC clamp is in boldface) and HDA2 (5’-GTA TTA CCG CGG CTG CTGGCA C-3’) [18]. PCR amplification was performed with the appropriate cDNA template, consisting of initial heating at 94°C for 4 min followed by 30 cycles of denaturing (94°C, 30 s), annealing (56°C, 30 s) and extension (72°C, 2 min) and a final extension at 72°C for 10 min. DGGE was performed using the Bio-Rad DCode Universal Detection System (Bio-Rad, Mississauga, ON, Canada) to separate PCR products according to their GC content. The gel contained 10% polyacrylamide (acrylamide/bisacrylamide 35.7:0.8) with a 35–65% gradient of urea and formamide increasing in the direction of electrophoresis (a 100% denaturing solution consisting of 7M urea and 40% deionized formamide). The electrophoresis was conducted in 1×TAE buffer under 100 V at 60°C for 16 h. DNA bands in gels were visualized by silver staining [19]. Images of the gel were taken by a Nikon Camera (Japan).

### Recovery of bands from DGGE gels and sequencing analysis

Specific bands of interest were excised from the DGGE gel as described [20]. The gel fragments were washed twice with standard PCR buffer (New England Bio-labs, Ipswich, MA, USA) and then incubated in diffusion buffer [the PCR buffer plus 0.1% Triton-X100] at 4°C overnight. The eluted DNA was re-amplified by PCR under the same conditions as described above and cloned into pCR4-TOPO (Invitrogen, Carlsbad, CA, USA). Clones were selected and subjected to DGGE to confirm that migration of the cloned products on DGGE were the same as the original band positions. After confirmation, the cloned PCR products were sequenced and the sequences were compared to 16S rRNA gene data base by BLASTn (GenBank, NCBI) to determine the closest sequence homologies for each band.

### Quantitative PCR (qPCR)

qPCR was performed using a Stratagene Mx3005p thermal cycler and iTaq SYBR Green Supermix with ROX (Bio-Rad, Mississauga, ON, Canada). Briefly, the program was 4 min at 95°C, then 40 cycles of 95°C for 30 s, 53–59°C for 30 s, and 72°C for 30 s. Fluorescence was measured after each annealing. The gene for 16S rRNA was used as an internal control [5]. Relative mRNA levels of genes of interest were determined and normalized to 16S rRNA using a modified 2^-ΔΔCt^ method [21]. The qPCR data are expressed as the changes in expression levels in AE lesion positive loops compared with the levels in AE lesion negative loops. To test whether the RNA isolated from the contents of pig intestine contained inhibitors of enzymatic reactions, an inhibition test was done as described previously [5].

### Statistical analysis

All analyses were performed with SAS for Windows version 8.02 (SAS Institute). Relative mRNA levels of genes of interest were similarly analysed for each treatment group of four to six biological replicates. Tukey’s test was used for multiple comparisons. *P* values ≤0.05 were considered significant.

## Results

### Colonization of *E*. *coli* O157:H7 in the pig ligated intestine

Of the 31 pigs, 26 pigs showed *E*. *coli* O157:H7 colonization (villi showing adherent bacterial clusters ≥ 5 bacteria). The adherent bacteria were confirmed to be *E*. *coli* O157 by immunohistochemistry and electron microscopy of sections with adherent bacteria showed typical AE lesions (data not shown).

The degree of colonization ranged from 0.86 to 78% (percentage of villi with adherent bacterial clusters ≥5 bacteria) with an average of 30.2 ± 20.2% (mean ± SD). Eight pigs with a high frequency of *E*. *coli* O157:H7 colonization (>20%) (AE positive) and 4 pigs with no *E*. *coli* O157:H7 colonization (AE negative) were chosen for RNA based DGGE analysis. The AE positive pigs were from 6 different experiments, and the 4 AE negative pigs were from 4 different experiments.

### DGGE fingerprinting comparison and sequence analysis

**Fig 1:**
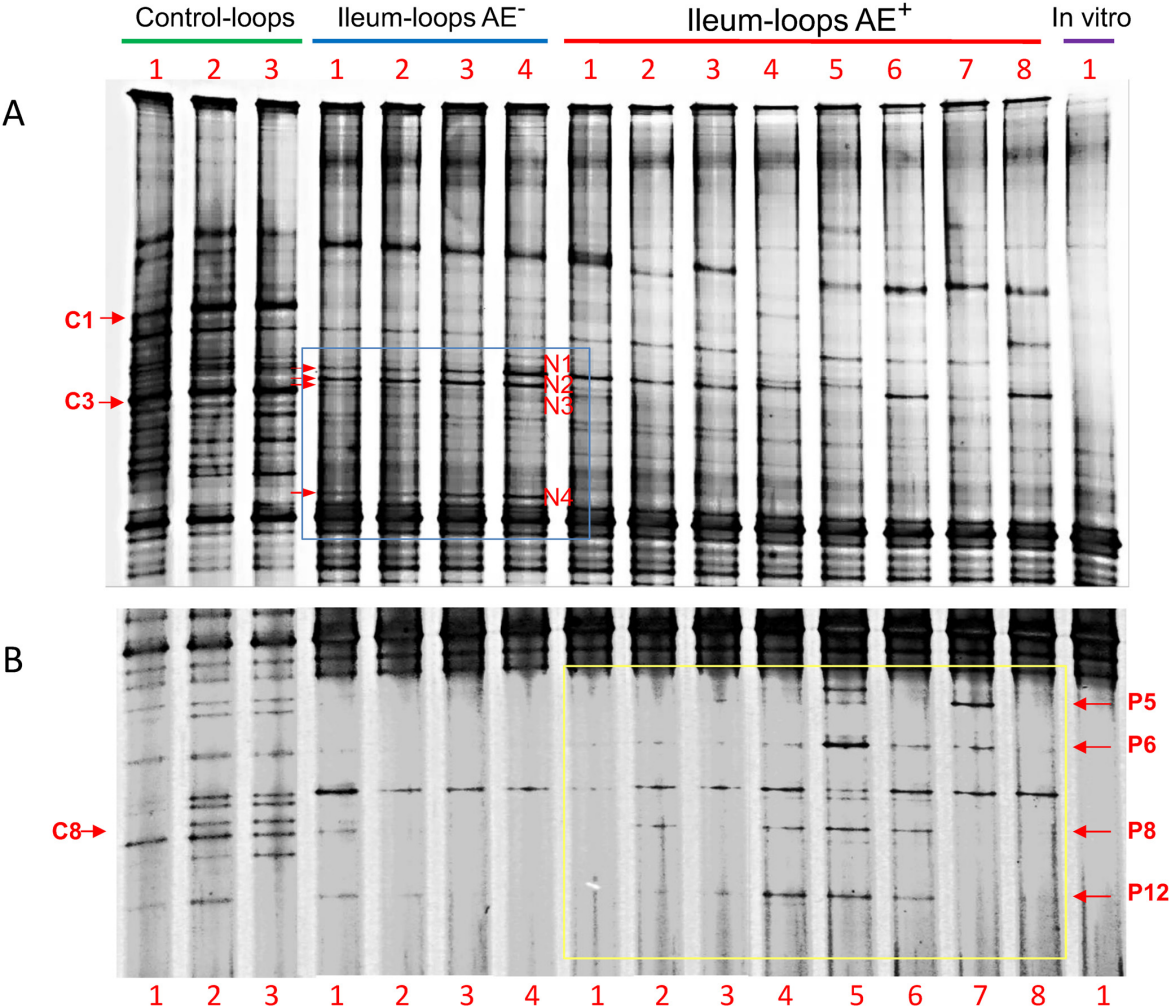
DGGE analysis of the bacterial community in the pig ileal loops with or without attaching and effacing (AE) lesion after inoculation of *E*. *coli* O157:H7 strain 86–24. The bacterial community profiles were generated by targeting 16S rRNA gene from reverse transcribed total intestinal bacterial RNA of the ileal contents. Figures A and B are overlapped top and lower portions of the same DGGE profiles but from different DGGE pictures with different resolutions. The profiles included control ileal loops inoculated with EMEM (control-loops), ileal loops without AE lesion and with AE lesion after *E*. *coli* O157:H7 inoculation (ileum-loops AE^-^ and ileum-loops AE^+^ respectively), and *E*. *coli* O157:H7 strain 86–24 grown *in vitro* before inoculation (In vitro).

The 16S rRNA gene fragment amplified from *E*. *coli* O157:H7 strain 86–24 grown *in vitro* and used for inoculation was run in parallel to the 3 samples from control pigs, followed by 4 samples from pigs without AE lesions and 8 samples from pigs with AE lesions. The control pigs were inoculated with EMEM with normal ileum [11]. The DGGE banding fingerprints showed differences in ileal microbiota population of AE-negative, AE-positive, and control pigs. The band intensity was visually examined. Among the highlighted differences, two bands were associated with AE negative pigs, band N2 and N4 (AE^-^ bands), which were present in all 4 AE negative samples and were present in either low intensity or absent from the control and samples from the AE-positive pigs (Fig 1). It is noteworthy that band N1 was present at higher intensity in both AE-negative and AE-positive pigs compared to the controls; while band N3 in samples from the AE-negative pigs showed a markedly decreased abundance compared with the controls (corresponding to band C3), and was almost not detected in 7 of the 8 AE-positive pigs. Several bands were more abundant in some AE-positive pigs compared to the AE-negative pigs, i.e. bands P5, P6, P8 and P12 (Fig 1). These bands were almost non-detectable in the AE negative pigs; although they were in similar abundance compared to the control pigs at the corresponding positions (Fig 1). However, some of these bands showed higher abundance than in the control, such as P5 in AE^+^ pig #7 and P6 in AE^+^ pig #5. The intensity of band P12 was higher in 3 of the 8 AE^+^ pigs than in both AE^-^ and control pigs (Fig 1). After challenge with EHEC O157:H7 strain 86–24, band C1, present in higher abundance in the control pigs, was sharply decreased in both AE^-^ and AE^+^ pigs. Band C8 migrated in the same position as band P8 (Fig 1).

Prominent bands identified on DGGE profiles were excised, cloned and sequenced to identify species of origin. Results of the sequence analysis by BLASTn are listed in Table 1. Bands N2 and N4 belonged to *Veillonella caviae* and uncultured *Bacteroides* sp. respectively. Bands N1 and N3 belonged to *Streptococcus* sp. and *Pasteurella aerogenes*, respectively. Bands C3 and C8 from the controls, with migration positions corresponding to bands N3 and P8, and were identical to these two bands, respectively, by sequence analysis (Table 1).

**Table 1:** Sequence analysis of bands associated with AE-positive and AE-negative ligated ileal loops of pigs inoculated with *E*. *coli* O157:H7.

*Band*	*Closest isolate relative*	*Identity (%)*	*Accession No*.
C1	*Haemophilus parasuis* strain AZ2-1	100	GU226388.1
N1	*Streptococcus* sp. 1561-2D2-04	100	FN908166.1
N2	*Veillonella caviae* strain PV1	99	NR_025762.1
N3/C3	*Pasteurella aerogenes* strain P592	99	AY465373.1
N4	Uncultured *Bacteroides* sp.	99	GU905761.1
P5	*Clostridium perfringens*	100	AB910734.1
P6	*Clostridium chauvoei* strain ASU55	99	KF372580.1
P8/C8	*Clostridium* sp. clone JCC	100	HG726039.1
P12	Uncultured *Clostridium* sp. Clone PLYFP96	99	JN792362.1

### Expression of virulence related genes in EHEC from AE positive and negative ileal loops

To understand the genetic basis that might lead to the different colonization and AE lesion phenotypes by EHEC O157:H7 strain 86–24 inoculated in pig ligated ileal loops, a set of 72 genes, including genes encoding LEE proteins and effectors, putative adhesins and virulence factors, and regulatory proteins, were evaluated by qPCR (S1 table) and compared between the EHEC from AE positive and negative ileal loops. The expression data are summarized in Table 2. A 3-fold change cutoff was considered as having a significant difference in bacterial gene expression provided there was statistical significance.

**Table 2:** Gene expression profiles of O157:H7 in pig intestinal loops as determined by qPCR.

Functional category and gene	Relative fold expression in intestinal loops		
	AE-negative loops	AE-positive loops	Control loops
**LEE genes**			
*tir*	1±0.54	18.11±13.5*	0.16±0.06*
*eae*	1±0.79	50.7±25.53*	0.13±0.06*
*ler*	1±0.73	4.54±2.38*	0.08±0.48*
*grlA*	1±0.60	4.08±3.24	0.04±0.03*
*grlR*	1±0.38	2.41±1.62	0.07±0.03*
*espD*	1±0.5	263.6±166.9*	0.05±0.01*
*espA*	1±0.33	210.76±91.52*	0.10±0.01*
**Toxin and putative toxin genes**			
*vt2A*	1±0.27	2.45±1.74	1.84±1.29
*vt2B*	1±0.40	1.99±1.40	0.98±0.6
*ehxA*	1±0.71	0.66±0.39	0.17±0.09
*stcE*	1±0.63	6.09±3.74*	0.38±0.34
*pagC*	1±0.55	1.61±1.00	0.26±0.23
*ent*	1±0.39	4.64±1.48*	0.29±0.13*
**Adhesins and putative adhesins**			
*aidA*_*15*_	1±0.28	9.34±5.03*	0.14±0.08*
*aidA*_*48*_	1±0.20	5.56±4.47	0.45±0.30
*iha*	1±0.13	6.95±4.44*	0.22±0.05*
*fliC*	1±0.98	13.49±5.51*	3.23±0.24
*toxB*	1±0.53	2.61±1.12	0.25±0.24
*eaeH*	1±0.28	4.78±3.23*	0.45±0.30
*efa1’-a*	1±0.62	2.74±1.06*	0.37±0.31
*efa1’-b*	1±0.29	1.56±0.95	0.35±0.26
*lpf*_*141*_	1±0.47	1.34±0.76	0.09±0.06*
*lpf*_*154*_	1±0.14	0.37±0.20*	0.11±0.08*
*fimA (pilin)*	1±0.15	0.26±0.18*	0.45±0.42
*hcpA*	1±0.55	1.27±1.07	2.18±1.13
*ompA*	1±0.21	9.33±3.28*	4.81±2.79
**Global regulatory genes**			
*himA*	1±0.43	5.49±2.03*	2.38±2.03
*hns*	1±0.60	6.50±3.13*	3.99±1.99
*hha*	1±0.54	1.74±1.40	0.94±0.36
*bipA*	1±0.97	4.37±1.56*	1.60±0.85
*fis*	1±0.96	7.57±4.15*	5.75±4.58
*lrp*	1±0.45	2.39±0.79	1.14±0.57
*phoQ*	1±0.99	1.34±0.65	0.94±0.68
*phoP*	1±0.92	1.44±0.53	1.09±1.05
*hfq*	1±0.30	2.36±0.83*	1.01±0.64
*etrA*	1±0.21	2.14±1.50	0.45±0.26
*eivF*	1±0.22	0.49±0.24*	0.39±0.24
**QS and its regulated genes**			
*luxS*	1±0.61	3.46±1.38*	3.3±1.8
*qseA*	1±0.81	1.09±0.54	0.41±0.33
*qseB*	1±0.53	3.24±2.83	1.81±1.57
*qseC*	1±0.44	2.27±1.25	1.48±1.16
*qseE*	1±0.37	2.41±1.94	0.72±0.56
*qseF*	1±0.11	3.57±2.03*	1.31±0.86
*flhD*	1±0.46	4.03±3.02	3.76±3.57
*kdpE*	1±0.71	2.72±1.84	2.58±1.34
*rcsB*	1±0.63	3.02±1.61	2.01±1.65
**Acid response and stress genes**			
*gadA*	1±0.22	2.40±0.78*	0.25±0.21*
*gadC*	1±0.34	4.85±4.80	0.26±0.26*
*gadE*	1±0.64	1.47±0.71	1.02±0.39
*gadX*	1±0.30	1.76±0.93	0.60±0.50
*gadW*	1±0.84	0.89±0.66	1.01±0.71
*adiA*	1±0.61	2.35±1.6	2.3±2.23
*ureC*	1±0.59	3.86±1.62*	0.49±0.47
*ureD*	1±0.50	4.97±2.51*	1.09±0.84
*mnmE*	1±0.30	2.04±1.29	3.19±1.64
*ydeO*	1±0.46	1.44±1.07	0.7±0.32
*evgA*	1±0.73	0.39±0.28	0.91±0.69
*katP*	1±0.25	2.80±2.10	0.05±0.04*
*sodA*	1±0.89	3.54±2.04	2.33±2.20
*chuA*	1±0.14	8.72±5.52*	0.77±0.25
*terC*	1±0.52	7.35±1.79*	0.37±0.14*
*terF*	1±0.18	8.39±4.95*	0.47±0.33
*rpoA*	1±0.33	7.21±2.60*	1.31±0.49
*rpoS*	1±0.40	6.35±4.41*	4.23±2.01
**Secreted proteins**			
*espP*	1±0.72	2.52±0.95	0.67±0.58
*espJ*	1±0.19	1.11±0.39	0.27±0.23*
*espFu*	1±0.48	1.82±0.81	0.74±0.34
*nleA*	1±0.38	14.5±5.59*	0.57±0.25
*nleB*	1±0.67	4.74±2.72*	0.34±0.3
*nleD*	1±0.41	1.72±1.11	0.04±0.04*
**Genes with unknown function**			
*yhbM*	1±0.20	2.73±1.97	0.78±0.51
*Z1006*	1±0.42	2.21±1.28	1.03±0.63
*Z3276*	1±0.87	1.54±1.24	0.57±0.30

^a^, Data are presented as relative fold expression (RFE) and represent the changes in extent of transcription compared to that of the bacteria from AE-negative loops (assigned a value of 1.0).

^b^, Data are expressed as the means ± SD for RNA extracted in 4–6 biological replicates.

^c^, Control loops were inoculated with EMEM medium only.

* indicates *p*<0.05 as compared to bacteria from AE-negative loops.

### 1. LEE island and toxin genes

Given that LEE genes are necessary for AE lesion formation and intimate attachment, we assessed the relative levels of LEE gene expression in EHEC that caused colonization and AE lesions in pig ligated ileal loops compared with those that did not. As expected, *eae* and *tir*, encoding the adhesin intimin and translocated intimin receptor (Tir), respectively, as well as the key regulator of LEE, *ler*, were increased by 50, 18 and 4.5 fold respectively in EHEC from AE lesion loops. The genes *espA* and *espD* were sharply increased by 210- and 260–fold, respectively, in EHEC from AE-positive loops.

The levels of transcripts for verotoxin genes *vt2*A and *vt2*B were not different in EHEC from AE positive and negative loops (Table 2). Of note, *ent*/*espL2*, located in O island (OI)-122, was increased in the EHEC from AE positive loops by ~4 fold (Table 2, *P*<0.05).

### 2. Putative adhesins, secreted proteins and stress genes

The gene encoding putative adhesin *ehaA*(*aidA*_*15*_), located in OI-15, was increased by ~9.3 fold in AE positive loops (Table 2, *P*<0.05). OI-48 genes, including putative adhesin *iha*, and genes involved in stress response *terC* and *ureD* were almost uniformly increased by approximately 5.0–7.3 fold, although *ureC* was increased by only 3.9-fold in AE positive loops (Table 2, *P*<0.05).

The *hcpA* gene encodes hemorrhagic coli pili (HCP), a type IV pilus (TFP) associated with EHEC O157 pathogenicity. Our data showed that the expression of *hcpA* gene was not different in EHEC O157:H7 from AE positive loops compared with EHEC O157:H7 from AE-negative loops (Table 2).

*fliC* transcripts were 13.5-fold greater in EHEC from AE-positive than in EHEC from AE negative loops (Table 2, *P*<0.05). Similarly, *ompA* transcripts were 9.3-fold higher in EHEC from AE positive loops (Table 2, *P*<0.05). There was no significant difference in expression of genes encoding putative adherence factors *toxB*, *efa1’*-a, *efa1’*-b, *lpf*_*141*_ and *lpf*_*154*_ between EHEC from AE-positive and AE-negative loops, but *fimA* transcripts showed a 3.8-fold decrease in AE-positive loops (Table 2).

The non-LEE encoded (Nle) effector gene *nleA* was sharply increased 14.5-fold and there was a significant increase in gene *nleB* (4.7-fold) in the AE-positive loops compared with AE-negative loops. Putative virulence gene *stcE* was elevated 6.1- fold (Table 2, *P*<0.05).

### 3. Regulatory genes

LEE genes and other virulence factors are tightly controlled by environmental cues and co-ordinately regulated by several regulatory systems such as global regulators and quorum sensing [9, 22], however, the importance of these regulatory systems *in vivo* are not clear. Expression of global regulators *rpoS*, *himA* (encoding IHF), *bipA*, *fis*, was significantly upregulated in EHEC from AE positive loops. Interestingly, transcripts for most quorum sensing genes were not significantly different in EHEC from both AE positive and negative loops, except *luxS* and *qseF*, which showed 3.5 and 3.6-fold increases (Table 2, *P*<0.05).

## Discussion

Enteric pathogens, including EHEC O157:H7, *Vibrio cholerae* and *Citrobacter rodentium*, have been reported to exhibit enhanced infectivity upon passage through host intestine [8, 23, 24]. The mechanism of the enhanced infectivity phenotype is not clear, however, available data suggest that the intestinal environment favors the expression and secretion of virulence factors for infection and AE lesion by EHEC in pigs (8). This may result in selection of bacteria that readily form AE lesions. As the intestinal microbiota comprises an important part of the host intestinal environment, alteration of the normal intestinal microbiota and the resulting microbiota-dependent activation of virulence gene expression may play a critical role in infectivity by enteric pathogens. It has been shown that *Salmonella* infection disrupted the microbial composition of a murine gastrointestinal tract and alteration of the microbiota was associated with increased host susceptibility to *Salmonella* infection in a mouse model [25, 26].

In the present study, we used RNA-based DGGE to compare the microbiota of the ileum with AE and without AE lesion. One advantage of using RNA-based microbiota analysis is that it identifies active members of the bacterial community, and changes in banding pattern and intensity in DGGE indicate changes in active microbial populations, potentially linking prominent microbial populations to specific functions [27, 28]. In the present study, we observed that AE-positive ileal loops resulting from inoculation of pig intestinal loops with EHEC O157:H7 strain 86–24 was associated with differences in gene expression of EHEC O157:H7 and in intestinal microbial composition compared with AE-negative ileal loops. Two DGGE bands belonging to *Veillonella caviae* and Bacteroides sp. were more prominent in AE^-^ loops compared to the controls, and were almost non-detectable in AE^+^ loops. Species of *Veillonella caviae* are considered probiotic. These species are lactate utilizers and have been used as probiotics for poultry due to their inhibitory activities against pathogens, such as *Listeria monocytogenes* and *Salmonella* Typhimurium [29, 30]. *Bacteroides* sp. such as *B*. *fragilis* and *B*. *vulgatus* have probiotic activities in relieving neurodevelopmental disorders and against *E*. *coli*-induced colitis in gnotobiotic mice respectively [31, 32]. A reduced level of intestinal *B*. *vulgatus* was suggested to be involved in intestinal inflammation in cystic fibrosis [33]. In our present study, we do not know whether the observed increase in activities of *Veillonella caviae* and *Bacterioides* sp. was a response to *E*. *coli* O157:H7 or might have existed before inoculation of the ileal loops. Therefore, a functional relationship between the colonization and virulence of *E*. *coli* O157:H7 and presence or activity of *Veillonella caviae* and *Bacterioides* sp. requires further investigation.

In AE^+^ pigs, in addition to a reduced population or activity of *Veillonella caviae* and the *Bacterioides* sp., some pigs appeared to have increased populations/activities of *Clostridium* sp. such as *C*. *chauvoei* and *C*. *perfringens*. *C*. *chauvoei* is known as a cause of blackleg in cattle and entercolitis in humans [34, 35] but is not associated with disease in pigs. However, our data showed that these organisms were also present in the control pigs with similar abundance/activity. It has been reported that there was a negative correlation between *C*. *perfringens* and *Lactobacillus* sp. populations in chicken intestine, the latter is an important normal intestinal microbiota of chicken [36]. The underlying mechanisms involved in the increases in bacteria associated with presence and absence of the AE lesion are not understood. Individual variations of pre-existing microbiota and host factors such as hormones may result in differential enhancement or inhibition of growth of certain members of the microbiota. It cannot be ruled out that pre-existing microbiota may be prone to alteration of microbiota populations in the presence of EHEC. Further studies are required to compare the microbial population before and after challenge of *E*. *coli* O157:H7 in the same animals.

Disturbance of the microbiota can alter the intestinal microbial metabolome, a collection of molecules of microbial origin with specialized functions and important physiological effects, such as hormones and thereby increase the susceptibility of the host to pathogen infection [37, 38] and change virulence gene expression of pathogens. Expression of virulence genes of EHEC O157:H7 86–24 from pig ileal loops was examined by qPCR and compared between EHEC from AE-positive and AE-negative pigs. As expected, expression of LEE genes was significantly increased in EHEC from AE positive pigs. In addition, expression of putative adhesins, EhaA and Iha and putative virulence factors StcE and NleA was also increased in EHEC from AE positive pig ligated ilea. These data suggest that in addition to LEE genes, these putative adhesins and virulence factors that were significantly up-regulated in the intestine might be involved in colonization and AE lesion phenotype. It is well known that EHEC LEE genes and other virulence factors are regulated both tightly and coordinately by environmental signals [5, 39]. However, it is not understood what intestinal signals are responsible for virulence gene upregulation and the resultant colonization and AE lesion phenotype. Among the regulatory factors, the present study showed that most QS gene expression was not changed in EHEC from AE-positive and AE-negative pigs, except *luxS* and *qseF* that were upregulated. QseF has been suggested to play a role in LEE gene activation and AE lesion formation [40, 41]. However, our data showed that expression of the genes *espFu*, *flhD* and *kdpE*, downstream of the quorum sensing QseE/F and QseC/B regulated genes, was not different between EHEC from AE positive and AE negative pigs (Table 2).

In summary, the present study demonstrated that there were differences in the intestinal microbiota in pig ligated ileal loops challenged with EHEC O157:H7. AE-negative pigs had increased activities/populations of *Veillonella caviae* and *Bacterioides* sp., and decreased populations of some *Clostridium* sp., suggesting that these alterations of intestinal microbiota might play a role in AE lesion development. Concomitantly, expression of LEE genes, putative adhesins, acid resistant genes *ureC/D* and quorum sensing genes *luxS* and *qseF* was increased in EHEC from AE-positive pigs. Further studies are required to understand how the microbiota was changed and the role of these organisms in the control of EHEC infection. Studies in which the microbiota is examined before inoculation of ligated pig ileal loops may also indicate whether the differences in the microbiota that were observed existed before or after inoculation of the loops.

## Supporting Information

S1 Table Primers used for quantitative PCR* and their target genes. *All the primers were designed in the present study unless referenced.
(XLS)
